# Photon-Counting Computed Tomography in Thoracic Surgery: A Narrative Review of Current and Future Applications

**DOI:** 10.3390/cancers17223656

**Published:** 2025-11-14

**Authors:** Giuseppe Mangiameli, Debora Brascia, Filippo Lococo, Giuseppe Marulli

**Affiliations:** 1 Division of Thoracic Surgery, IRCCS Humanitas Research Hospital, Via Manzoni 56, Rozzano, 20089 Milan, Italy; debora.brascia@hunimed.eu (D.B.);; 2 Department of Biomedical Sciences, Humanitas University, Via Rita Levi Montalcini 4, Pieve Emanuele, 20090 Milan, Italy; 3 Thoracic Surgery Unit, Università Cattolica del Sacro Cuore, Largo F. Vito 1, 00168 Rome, Italy; 4 Thoracic Surgery Unit, Fondazione Policlinico Universitario A. Gemelli IRCCS, 00168 Rome, Italy

**Keywords:** photon-counting CT, thoracic surgery, lung cancer, spectral imaging, surgical planning

## Abstract

Photon-counting computed tomography (PCCT) introduces a new era in thoracic imaging by offering ultra-high spatial resolution, reduced noise, spectral imaging capabilities, and lower radiation dose compared to conventional CT. These features are particularly relevant in thoracic surgery, where precise anatomical and functional assessment is essential throughout the perioperative period. This narrative review outlines the clinical potential of PCCT in surgical planning, intra- and postoperative evaluation, and follow-up of both oncologic and non-oncologic thoracic conditions. PCCT enables accurate bronchovascular mapping and iodine-based perfusion imaging, supporting sublobar resection planning and risk stratification in patients with complex anatomy or reduced lung function. Postoperatively, it enhances detection of subtle complications—such as air leaks or hematomas—and improves image quality near metallic implants through advanced artifact reduction techniques. The ability to combine high-resolution imaging with functional data allows for comprehensive evaluation in a single scan and may aid in differentiating fibrosis from local recurrence. Despite its promises, PCCT adoption is currently limited by high cost, restricted availability, and the need for training and system integration. Furthermore, prospective clinical studies are still needed to determine its impact on surgical outcomes. As technological and infrastructural challenges are addressed, PCCT may become a valuable component of image-guided thoracic surgery, contributing to safer, more personalized care.

## Simple Summary

Photon-counting computed tomography (PCCT) is an innovative CT technology that detects individual X-ray photons, producing images with much higher sharpness and less noise than conventional CT, while also reducing radiation exposure. These features make PCCT particularly valuable in thoracic surgery, where precise imaging is crucial before, during, and after an operation. This review summarizes how PCCT can improve surgical planning, intraoperative guidance, and postoperative follow-up in patients with thoracic diseases. By providing detailed three-dimensional views of airways and blood vessels, PCCT supports accurate and safer lung resections. It also helps detect early postoperative complications and differentiates scar tissue from tumor recurrence in follow-up scans. Although its use is currently limited by high costs and the need for specialized training, PCCT has the potential to transform the way thoracic surgeons plan and monitor their procedures, contributing to safer and more personalized surgical care.

## 1. Introduction

In an era of widespread lung screening, early lung cancer detection and increased indications for anatomical lung segmentectomy, precision imaging has become a cornerstone of safe and effective thoracic surgery. High-resolution computed tomography (HRCT) has long been the standard for preoperative planning and postoperative monitoring. However, conventional CT systems based on energy-integrating detectors (EIDs) present intrinsic limitations such as suboptimal spatial resolution, limited material differentiation, and metal artifacts that may compromise image interpretation in postoperative settings [1,2]. Additionally, cumulative radiation exposure remains a major concern, particularly for oncologic patients requiring long-term imaging follow-up or lung cancer screening programs [3,4]. In this scenario, the advent of PCCT marks a major technological advancement, enhancing diagnostic accuracy, expanding the clinical utility of thoracic imaging, and addressing many of the limitations inherent to traditional CT [5].

Unlike conventional detectors, PCCT utilizes photon-counting technology that detects individual X-ray photons along with their energy levels. This results in enhanced spatial resolution, inherent spectral imaging capabilities, improved signal-to-noise ratio, and a substantial reduction in radiation dose [6]. These improvements may be particularly relevant for thoracic surgeons, who require detailed anatomical and functional information to guide clinical decisions, either for preoperative planning, intraoperative navigation, and postoperative evaluation, especially in this new era of precision and tailored thoracic surgery.

Although the role of PCCT has been increasingly studied in radiology—particularly in cardiovascular and oncologic imaging—its specific implications in thoracic surgery remain underexplored. The aim of this narrative review is to bridge the gap between radiologic innovation and surgical application, providing thoracic surgeons with a practical overview of how PCCT may enhance perioperative care, by analyzing four main domains of thoracic oncology (Table 1): (a) diagnosis of lung nodules, (b) preoperative planning, particularly in segmentectomy, (c) intra- and postoperative assessment and (d) non-oncologic thoracic conditions.

## 2. Technical Overview of PCCT

PCCT marks a substantial advancement in CT imaging technology, offering significant technical advantages over conventional energy-integrating detector (EID) systems— particularly relevant in thoracic surgery, where accurate tissue characterization, high-resolution imaging, and effective metal artifact reduction are essential.

**Figure 1:**
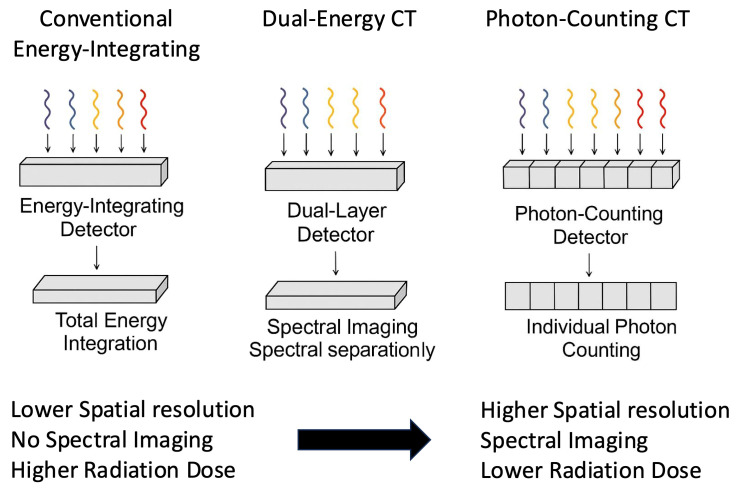
Comparative schematic representation of Photon-Counting CT (PCCT), Dual-Energy CT (DECT), and Conventional Energy-Integrating Detector CT (EID-CT).

In contrast to conventional energy-integrating detector (EID) CT and dual-energy CT (DECT), PPCT achieves spectral separation and high spatial resolution simultaneously within a single acquisition (see Figure 1). Unlike DECT, which derives spectral information from two different X-ray spectra, PCCT directly measures the energy of each photon, providing more accurate material characterization and minimizing temporal misregistration [6,7,8]. These features, combined with reduced noise and smaller detector elements, represent a substantial technological evolution over current state-of-the-art CT systems and have direct implications for thoracic surgical imaging, particularly for surgical planning and postoperative evaluation.

A quantitative comparison of the main technical parameters between PCCT, dual-energy CT, and conventional energy-integrating detector CT systems is summarized in Table 2.

### 2.1. Improved Spatial Resolution

One of the most remarkable advantages of PCTT is its ultra-high spatial resolution, made possible by smaller detector pixel sizes and the absence of light diffusion during signal conversion. Unlike energy-integrating detector (EID) systems, which rely on scintillators and suffer from interpixel crosstalk, photon-counting detectors (PCDs) directly convert X-ray photons into electrical signals. This architecture allows for voxel sizes as small as 0.1 mm, resulting in sharper image detail and improved delineation of fine anatomical structures. This enhanced resolution is particularly impactful in thoracic surgery, since it may facilitate the detection and characterization of (a) small pulmonary nodules, including pure ground-glass or part-solid lesions; (b) interstitial changes, such as reticulations or early fibrotic patterns, which are crucial in assessing operability and postoperative risk in patients with underlying lung disease; (c) distal bronchi and subsegmental vessels, which are essential in planning segmentectomy or complex sublobar resections.

Bartlett et al. [9] demonstrated that PCCT significantly improves nodule margin definition and internal texture analysis compared to conventional CT, even at lower radiation doses. This higher fidelity allows for more confident surgical planning, particularly in early-stage lung cancer, where millimetric margins can define resectability or extent of resection [9].

Moreover, in follow-up imaging, the ability to detect subtle volumetric changes—such as interval growth of subcentimetric nodules or early parenchymal alterations near surgical margins—may support earlier diagnosis of recurrence or complications.

### 2.2. Enhanced Contrast-to-Noise Ratio (CNR)

The combination of reduced electronic noise and inherent energy discrimination in PCCT improve the contrast-to-noise ratio, allowing for improved visualization of soft tissues and vascular structures while maintaining a lower radiation dose [7,10]. This direct photon-to-signal conversion not only minimizes background electronic noise, but also enables more accurate differentiation between tissues of similar density.

### 2.3. Spectral Imaging and Material Decomposition

The intrinsic spectral imaging capabilities of PCCT enable material decomposition and the generation of virtual monoenergetic images (VMIs), iodine maps, and virtual non-contrast reconstructions. These reconstructions provide both anatomical and functional insights within a single acquisition, offering clear benefits in thoracic oncology and postoperative assessment.

In current clinical practice, three spectral products are particularly valuable.

(i) Iodine maps provide quantitative surrogates of tissue vascularity and regional perfusion within a single acquisition, aiding in the differentiation between viable tumor and post-treatment fibrosis—a common diagnostic dilemma in surgical follow-up [11].
(ii) Virtual monoenergetic images (VMIs)—low-keV VMIs (≈40–60 keV) maximize iodine conspicuity and are helpful for detecting subtle enhancing foci (e.g., early recurrence or active inflammation) [11].
(iii) Effective atomic number (Z_eff) maps—an emerging tool for material characterization that may complement iodine maps when differentiating soft-tissue components; early experiences are promising, but thoracic outcome data remain limited compared with VMIs and iodine maps [9,10,11].

The ability to extract multiple image sets from a single scan—without the need for separate contrast phases or additional radiation—enhances efficiency and reduces patient risk. Moreover, the precision of spectral data supports quantitative imaging approaches and may serve as the basis for future radiomics or AI-based risk stratification tools.

### 2.4. Lower Radiation Exposure

Thanks to more efficient photon utilization and reduced electronic noise, PCCT can maintain or even improve image quality at significantly lower radiation doses compared to conventional CT. Several studies have reported dose reductions of up to 30–40% without compromising diagnostic accuracy in thoracic imaging [10,12]. This advantage is particularly relevant in lung cancer screening programs.

### 2.5. Metal Artifact Reduction

PCCT reduces beam-hardening artifacts from metallic implants through its intrinsic spectral capabilities and advanced reconstruction algorithms. This improvement enhances postoperative image interpretation in patients with sternal wires, surgical clips, or vascular prostheses, allowing clearer visualization of periprosthetic tissues and surgical sites [7,8].

## 3. Clinical Applications in Thoracic Surgery

With its unique combination of ultra-high spatial resolution, spectral imaging capabilities, and reduced radiation exposure, PCCT is poised to impact multiple aspects of thoracic surgical care. PCCT has the potential to enhance surgical care across the entire perioperative continuum including pre-, intra- and postoperative/follow-up (Table 1).

### 3.1. PCCT: From Lung Nodule Detection to Nodal Staging

Accurate evaluation of pulmonary nodules is a critical step in the management of thoracic surgical patients. Although conventional CT remains a cornerstone in clinical practice, it presents limitations in differentiating between benign and malignant lesions—especially in the case of small, subsolid, or partially calcified nodules. In this scenario, PCCT offers significant advantages, building on the technical improvements discussed above, by enabling more accurate evaluation of margins, internal structure, and density. Mohammadzadeh et al. recently published a systematic review and meta-analysis comparing PCCT with conventional EID-CT for the evaluation of pulmonary nodules. Their results, based on a total of 13 studies including 718 patients and 362 lung nodules, showed that PCCT achieved significantly higher image quality based on the Likert score, while maintaining a lower radiation exposure. The authors concluded that integrating PCCT into routine clinical workflows and lung cancer screening programs may enhance nodule detectability and improve diagnostic accuracy [13]. Similar findings were reported in a preliminary study by Hop et al., who evaluated a large field-of-view spectral photon-counting CT (SPCCT) prototype. Their results indicated that low-dose PCCT offers the potential to reliably detect and volumetrically assess pulmonary nodules, even with radiation dose reductions of up to 90% [14]. Additional evidence from a preclinical study by Kopp et al. supports these observations, demonstrating that a prototype PCCT system provided improved spatial resolution and soft-tissue contrast compared to conventional CT in pulmonary imaging, thereby enhancing visualization of small anatomical structures relevant to nodule detection and characterization [15]. Phantom studies comparing PCCT to dual-layer CT have shown a 47% average reduction in noise magnitude and significantly higher detectability indices for both solid and ground-glass nodules, confirming the objective gain in contrast efficiency [16].

In addition to morphological assessment, the enhanced spatial resolution of PCCT allows for the identification of smaller nodules and subtle volumetric changes over time—an aspect particularly relevant for lung cancer screening and follow-up of indeterminate lesions.

In parallel, spectral data also provide the potential for quantitative tissue analysis through material decomposition, such as iodine, calcium, and lipid mapping—which may facilitate the distinction between calcified, lipid-rich, or fibrotic components, an area of growing interest in risk stratification [17]. Combined, these features may improve the non-invasive differentiation between malignant and benign nodules, potentially minimizing the need for invasive diagnostic procedures.

Notably, PCCT also holds promise in the evaluation of tumor vascularity and perfusion. Through iodine mapping and dual-energy material quantification, PCCT can provide surrogate measures of tissue vascularization [18]. This may assist in distinguishing hypervascular malignant lesions from hypovascular benign nodules and could potentially support early detection of aggressive tumor lesions. Perfusion-sensitive imaging may also improve assessment of therapeutic response following systemic or ablative treatments.

Beyond nodule characterization and perfusion assessment, PCCT may also impact oncologic staging. While current literature on mediastinal staging with PCCT is limited, preliminary data from spectral imaging approaches suggest enhanced visualization of mediastinal structures and lymph nodes, potentially improving staging accuracy [19,20].

The combination of high spatial resolution and spectral imaging enables better delineation of nodal margins, internal architecture, and subtle enhancement patterns. Unlike conventional CT, which primarily relies on size-based criteria, PCCT may offer functional information that may enhance specificity in detecting metastatic involvement, thereby improving the accuracy of N-staging. Moreover, its ability to suppress artifacts near vascular and mediastinal structures supports more confident image interpretation, particularly in both preoperative planning and re-operative scenarios.

While the current standard for mediastinal staging includes PET/CT and invasive procedures such as EBUS-TBNA and mediastinoscopy, PCCT may offer a non-invasive alternative with advantages in spatial resolution and radiation dose. However, its diagnostic accuracy in this setting requires validation through prospective studies before routine clinical adoption.

### 3.2. Preoperative Surgical Planning: Functional and Structural Planning with PCCT

In thoracic surgery, accurate preoperative planning has become fundamental, particularly with the increasing use of anatomical segmentectomies for early-stage non-small cell lung cancer (NSCLC). As sublobar resections see growing adoption for preserving pulmonary function without compromising oncologic outcomes, meticulous bronchovascular mapping and thorough evaluation of anatomical variations are essential to ensure precise segmental resection and adequate margins. Advanced imaging techniques, including three-dimensional (3D) reconstructions, now play a pivotal role in guiding surgical strategy and minimizing intraoperative uncertainty. Hojski et al. [21] reported that 3D reconstructions obtained from high-resolution CT significantly decrease the rate of conversion to thoracotomy and reduce major complications during VATS segmentectomy. Similarly, data from the multicentric DRIVATS study [22] indicate that CT-based 3D planning improves intraoperative outcomes and decreases complications in VATS segmentectomy.

PCCT meets the need for more detailed anatomical imaging by offering higher resolution, less noise, and better contrast, especially for vessels and bronchi, compared to standard CT. These technical advancements enable direct, high-fidelity visualization of small pulmonary vessels and airways. In addition, PCCT-generated datasets can be utilized for advanced 3D structural reconstructions, facilitating accurate anatomical mapping that enhances both preoperative planning and intraoperative navigation. Kerber et al. demonstrated that PCCT-derived iodine maps can reliably identify perfusion defects with diagnostic accuracy comparable to SPECT/CT, but with significantly lower radiation exposure [23,24].

Moreover, high-resolution datasets derived from PCCT can be effectively integrated into augmented reality (AR) platforms and intraoperative navigation systems, enhancing real-time anatomical guidance, as already demonstrated with conventional CT in previous clinical experiences [25,26].

### 3.3. Preoperative Surgical Planning: Functional Planning with PCCT

Beyond its morphological advantages, PCTT enables simultaneous evaluation of pulmonary function within a single acquisition. PCCT facilitates accurate volumetric analysis of the residual lung parenchyma, supporting preoperative functional assessment, especially in patients with emphysema or limited pulmonary reserve, where functional planning is as critical as anatomical precision [27,28]. In this context, PCCT is also emerging as a valuable tool for preoperative risk stratification. Its high-resolution tissue characterization enables precise quantification of parenchymal diseases—such as emphysema and fibrosis—and supports operability assessment, outcome prediction, and surgical decision-making through imaging biomarkers. Iodine maps derived from PCCT can serve as high-resolution surrogates of pulmonary perfusion, accurately depicting segmental and subsegmental perfusion defects in conditions such as pulmonary embolism or hypoperfusion secondary to emphysema. However, prospective studies are needed to confirm whether PCCT-based functional planning translates into measurable surgical outcomes. Beyond surgical planning, PCCT also plays a pivotal role in the intra- and postoperative phases, supporting early complication detection and long-term surveillance.

### 3.4. Redefining Postoperative Imaging in Thoracic Surgery: The Role of PCCT

PCCT offers several advantages in the postoperative evaluation of thoracic surgical patients. Its ultra-high spatial resolution and spectral imaging capabilities enhance the detection of common postoperative complications, such as air leaks, small pleural or parenchymal fluid collections, and intrathoracic hematomas. Prompt identification of these abnormalities may support timely clinical interventions, potentially reducing postoperative morbidity, duration of chest drainage, and length of hospital stay [29].

PCCT provides precise assessment of bronchial stump integrity, which is critical in patients undergoing pneumonectomy or presenting with risk factors for impaired healing. Its superior contrast resolution and high-definition anatomical detail enable accurate detection of subtle abnormalities, such as small fistulas or early signs of dehiscence, and support the identification of residual or recurrent disease, even in the presence of postoperative fibrosis and inflammatory changes [30].

Through spectral data acquisition and advanced reconstruction techniques such as virtual monoenergetic imaging (VMI) and metal artifact reduction algorithms (e.g., iMAR), PCCT enhances evaluation of bronchial stumps, mediastinal structures, and pleural spaces.

Several studies have demonstrated that VMI reconstructions at 110–130 keV combined with iMAR algorithms can significantly reduce blooming and streak artifacts, improving confidence in postoperative radiologic evaluation by allowing (a) a more accurate postoperative assessment, particularly in detecting residual collections, dehiscence, or recurrence near hardware; (b) improved planning for re-operations, where previous metallic implants may otherwise obscure critical anatomy and (c) enhanced interventional guidance, especially when evaluating drain placement or image-guided biopsies in artifact-prone regions [31,32].

Although PCCT has demonstrated improved metal artifact reduction compared with conventional energy-integrating CT, this benefit remains highly dependent on acquisition and reconstruction parameters, including tube potential, VMI energy selection, and energy-threshold configuration. While its enhanced spectral purity, reduced electronic noise, and use of high-energy bins contribute to mitigating streak and beam-hardening artifacts, advanced iterative MAR/iMAR algorithms implemented on modern CT systems already provide substantial artifact suppression. As a result, the incremental advantage of PCCT may be modest unless optimized VMI reconstructions or combined MAR strategies are applied. Its superiority over state-of-the-art iterative techniques therefore appears context-dependent and requires further standardized, comparative evaluation.

In long-term postoperative surveillance, the reduced radiation exposure of PCCT is particularly advantageous [33]. By integrating anatomical and functional information, PCCT enhances detection of subtle parenchymal changes, local recurrences, and metachronous lesions. This supports its role as a valuable tool in the longitudinal management of post-surgical lung cancer patients [6].

Distinguishing fibrosis from tumor recurrence near the resection margin is a common challenge in postoperative follow-up, as both may appear similar on conventional CT. PCCT’s spectral capabilities can help differentiate enhancing, vascularized recurrences from avascular fibrotic tissue, potentially improving specificity in oncologic surveillance [34].

Beyond diagnostic accuracy, the enhanced anatomical detail of PCCT supports interventional decision-making, such as image-guided drainage and bronchoscopic planning in suspected stump complications [35]. Additionally, its functional capabilities—like regional perfusion and aeration mapping—enable objective monitoring of recovery, including atelectasis resolution, compensatory hyperinflation, and persistent dysfunction [36].

In summary, PCCT improves intra- and postoperative assessment by combining high-resolution imaging, artifact reduction, and functional data. Its benefits are especially relevant after complex resections or during long-term follow-up, though prospective studies are needed to confirm its clinical impact and integration into care pathways.

### 3.5. Non-Oncological Thoracic Diseases

Beyond oncologic applications, PCCT is rapidly demonstrating added value in non-oncologic thoracic conditions, such as interstitial lung disease (ILD), pulmonary vascular disorders, and airway diseases. Thanks to a unique combination of ultra-high spatial resolution, multi-energy data acquisition, and dose efficiency, PCCT enables detection and quantification of subtle abnormalities previously difficult to assess.

#### 3.5.1. Interstitial Lung Diseases

In a recent study, Gaillard et al. [37] compared parenchymal analysis in 112 patients with ILD using ultra-high-resolution (UHR) images acquired with a PCCT scanner and high-resolution (HR) images obtained with an EID-CT. The authors concluded that the UHR scanning mode of PCCT enabled a more accurate depiction of early interstitial alterations in ILD, such as reticulations, micronodules, ground-glass opacities, bronchiectasis, and honeycombing, and allowed for reclassification of ILD patterns, while achieving a significant radiation dose reduction of approximately 27–32%. The UHR mode provides improved delineation of subtle fibrotic abnormalities, with the potential to impact diagnostic categorization of ILD subtypes.

The role of PCCT in the quantitative assessment of pulmonary fibrosis within machine learning models has recently been investigated. In a study comparing same-day conventional CT and PCD-CT in 52 patients with suspected ILD, quantitative analysis demonstrated good-to-excellent concordance across most imaging features, with the exception of honeycombing, which was more accurately delineated by PCD-CT. Notably, PCD-CT showed stronger correlations with pulmonary function tests for honeycombing, suggesting improved diagnostic performance, although model recalibration may still be required [38].

#### 3.5.2. Pulmonary Infections and Empyema

PCCT has shown significant advantages in the assessment of pulmonary infections by enhancing the visualization of consolidations, cavitations, and abscesses, as well as by providing functional information through iodine-based spectral imaging, which helps differentiate viable from necrotic tissue and guide interventional procedures [39]. In the specific context of pleural empyema, a retrospective study by Jungblut et al. [40] demonstrated that PCD-CT with low-energy virtual monoenergetic images (40 keV) achieves superior diagnostic accuracy (96%) and confidence compared to conventional CT. Although iodine maps did not enhance detection at low keV, they proved valuable at higher energies, particularly in cases of reduced contrast. These findings support the clinical utility of PCCT in improving the evaluation and management of infectious thoracic conditions.

#### 3.5.3. Pulmonary Embolism and Vascular Anomalies

PCCT is particularly valuable in assessing both acute and chronic pulmonary embolism. In a prospective study, Pannenbecker et al. [41] demonstrated that ultra-high-pitch, free-breathing CT pulmonary angiography (CTPA) using a photon-counting detector provides significantly superior image quality compared to standard EID-CT. PCCT demonstrated superior visualization of small emboli and improved delineation of vascular lumens, owing to higher spatial resolution and advanced noise-reduction algorithms. In the chronic setting, Kerber et al. [42] showed that PCD-CT iodine maps allow for accurate and low-dose diagnosis of chronic thromboembolic pulmonary hypertension (CTEPH), with high sensitivity (94%) and specificity (84%), based on imaging alone. Additionally, iodine-based perfusion maps derived from spectral data provide functional insights that can confirm perfusion defects corresponding to embolic obstruction. This functional–anatomical correlation enhances diagnostic confidence, potentially reducing the need for additional nuclear medicine studies like ventilation–perfusion (V/Q) scans or SPECT/CT [42]. Beyond embolic disease, PCCT also shows promise in detecting and characterizing pulmonary vascular anomalies such as arteriovenous malformations (AVMs), anomalous venous drainage, and congenital vascular variants. Spectral PCCT enables precise assessment of complex vascular anatomy, supporting identification of even subtle abnormalities while minimizing artifacts near metallic devices or calcifications. This is particularly valuable in preoperative planning, screening of hereditary hemorrhagic telangiectasia and follow-up of treated vascular lesions [42].

#### 3.5.4. Tracheobronchial Diseases

PCCT’s ultra-high spatial resolution enables detailed visualization of the trachea and major bronchi, which is particularly beneficial in patients with tracheal stenosis, malacia, or post-intubation injury. In a study by Milos et al. [43], different reconstruction settings for ultra-high-resolution PCCT of the lungs were compared. The BI64 kernel with 0.4 mm slice thickness provided the best image quality, significantly improving the visualization of the bronchial tree without compromising other anatomical structures. These findings support the reliability of this reconstruction setting for bronchial evaluation and its value in pulmonary imaging optimization.

The ability to depict subtle structural abnormalities—such as mural thickening, luminal narrowing, or airway collapse—facilitates diagnosis, preoperative planning, and procedural decisions such as tracheal resection or stenting. These capabilities are particularly valuable in complex airway reconstructions and in managing post-COVID airway complications.

#### 3.5.5. Thoracic Trauma

In the context of thoracic trauma, PCCT improves the detection of subtle rib fractures, lung contusions, pneumothorax, and small hemothorax, while minimizing radiation exposure.

In a recent study, Kaatsch et al. demonstrated that low-keV virtual monoenergetic images (40–50 keV) from PCD-CT significantly improve the differentiation between lung injury and atelectasis in polytraumatized patients, offering superior contrast and discriminability [44].

The use of low-energy virtual monoenergetic reconstructions enhances contrast between blood, fluid, and aerated lung, which can aid in distinguishing contusions from atelectasis—an essential factor in trauma triage and surgical decision-making.

#### 3.5.6. Transplant Imaging

In the post-transplant setting, PCCT may offer specific advantages in radiological surveillance. In a recent study published by Milos et al., ultra-low-dose PCCT was compared with ultra-low-dose spiral CT for post-transplant surveillance in 82 lung transplant recipients (41 vs. 41). The authors demonstrated that ultra-low-dose PCCT enables safe surveillance by preserving diagnostic image quality (e.g., for bronchiectasis and ground-glass opacities) while delivering less than 20% of the standard radiation dose (~0.3 mSv vs. ~1.4 mSv) [45]. These findings support the use of PCCT for monitoring chronic lung allograft dysfunction and infectious or fibrotic complications in a patient population particularly vulnerable to cumulative radiation exposure.

## 4. Limitations and Barriers to Implementation

Despite growing interest in PCCT and its promising applications in thoracic surgery, several barriers still limit its widespread adoption. The main challenges concern cost, availability, training, clinical validation, and integration into surgical workflows. PCCT systems require significantly higher capital investment and maintenance costs than conventional CT scanners, with units priced between USD 2.5 and 3 million—compared to USD 1.0–1.5 million for high-end EID-CT systems [6,46]. These financial constraints, coupled with limited availability—currently restricted to a few academic centers (e.g., fewer than 5 systems in clinical use in Italy as of 2024)—hamper broader implementation and equitable access [5].

Another critical aspect is the learning curve in interpreting PCCT-specific outputs, such as spectral data, iodine maps, and virtual monoenergetic images. Without adequate training, radiologists may underuse or misinterpret this information, diminishing diagnostic value [47]. Incorporating PCCT into radiology curricula and multidisciplinary training may help address this gap. Moreover, most current evidence comes from small or single-center studies focused on feasibility and image quality. Large-scale, multicenter trials are needed to validate the clinical impact of PCCT in thoracic indications—particularly in surgical planning and postoperative monitoring [48].

Furthermore, as this work is a narrative rather than a systematic review, it is inherently subject to potential selection bias and limited by the strength and heterogeneity of the available evidence. Most of the studies discussed are small, single-center, and preliminary in nature, often focusing on feasibility and image quality rather than clinical outcomes. Therefore, the conclusions drawn should be interpreted with caution and considered hypothesis-generating rather than definitive.

Finally, integration into surgical platforms remains limited. Although PCCT provides detailed 3D and functional reconstructions, interoperability with navigation systems, AR platforms, or robotic workflows is still suboptimal. Standardization of data formats and software compatibility will be essential to unlock its full potential [49].

In summary, while PCCT represents a major technological advancement, its successful integration into thoracic surgical practice will depend on addressing key limitations in cost, training, evidence generation, and system integration.

## 5. Conclusions

PCCT represents a significant advancement in thoracic imaging, offering unprecedented spatial resolution, reduced image noise, and integrated spectral capabilities within a single acquisition. These features enhance anatomical visualization, functional assessment, and artifact reduction—key elements for surgical planning, intraoperative guidance, and postoperative follow-up with direct implications for thoracic surgical practice.

In thoracic surgery, PCCT shows particular promise in refining anatomical segmentations, identifying perfusion defects, differentiating fibrotic scars from tumor recurrence, and supporting interventional decisions. Its ability to deliver both morphological and quantitative data in a low-dose, high-fidelity format suggests a paradigm shift toward more personalized and image-guided surgical care.

However, to ensure its broad clinical adoption, key barriers must be addressed—including cost-effectiveness, system integration, and user training. Importantly, large-scale prospective studies are still needed to confirm whether the technical advantages of PCCT translate into improved patient outcomes across the surgical continuum.

Given the narrative nature of this review and the predominance of preliminary single-center data, the conclusions presented should be regarded as exploratory and reflective of the current state of early clinical experience rather than conclusive evidence.

If these challenges are met, PCCT may soon become an integral component of thoracic surgical practice, bridging the gap between diagnostic imaging and procedural precision.

## Figures and Tables

**Table 1:** Clinical Applications of PCCT in Thoracic Surgery.

Clinical Domain	PCCT Advantage	Surgical Relevance
Diagnosis		
Lung nodule evaluation	High-resolution morphology, material decomposition	Accurate malignancy prediction
Tumor staging	Precise lymph node assessment, vascular invasion detection	Improved resectability evaluation
Preoperative planning		
Segmentectomy planning	3D visualization of vessels and bronchi	Safe and tailored resections
Postoperative assessment		
Postoperative follow-up	Artifact reduction, detection of small collections or air leaks	Early detection of complications
Non-oncologic thoracic conditions		
Interstitial lung disease (ILD)	Better parenchymal detail, quantitative analysis	Pre-op risk stratification, especially in CPFE
Metallic implants/sutures	Reduced blooming artifacts	Clear visualization in re-operations
Radiation dose	Up to 40% reduction vs. conventional CT	Safer follow-up protocols, especially in oncology

PCCT = Photon-Counting Computed Tomography; ILD = Interstitial Lung Disease; CPFE = Combined Pulmonary Fibrosis and Emphysema; CT = Computed Tomography.

**Table 2:** Comparative technical characteristics of photon-counting CT (PCCT), conventional energy-integrating detector (EID) CT, and dual-energy CT (DECT) systems.

Parameter	Conventional EID-CT	Dual-Energy CT (DECT)	Photon-Counting CT (PCCT)
Detector pixel size	~0.5–0.625 mm	~0.4–0.6 mm	~0.25–0.3 mm
Spatial resolution (line pairs/cm)	20–25	25–30	35–40
Energy discrimination	None	Dual spectra (dual-source or kVp-switching)	True multi-energy (per-photon energy binning)
Contrast-to-noise ratio (CNR)	Baseline	~1.2 × EID	~1.5–2 × EID
Noise characteristics	Electronic noise included	Reduced	Minimized (direct conversion)
Metal artifact reduction	Limited	Moderate	Enhanced

The following sections outline the main technical advantages of PCCT and their specific relevance in thoracic surgery.
